# A global perspective for managing obesity and improving health: conventional treatment and surgical options: 4th Annual Obesity Summit, London, April 2016

**DOI:** 10.4155/fsoa-2016-0059

**Published:** 2016-09-29

**Authors:** Adeel Nazir Ahmad, Kimberley L Edwards

**Affiliations:** 1 Department of Family Medicine, King Faisal Specialist Hospital & Research Centre, Jeddah, Saudi Arabia; 2 School of Medicine, University of Nottingham, Nottingham, UK

**Keywords:** euroscicon, obesity, obesity research, obesity summit, obesity surgery

## Abstract

**4th Annual Obesity Summit, London, 12–14 April 2016**

There are more than 1.9 billion overweight people worldwide, culminating in high rates of Type 2 diabetes; and cardiovascular, digestive and other health problems. This makes obesity a startling phenomenon and a significant global health epidemic. To address this, The 2016 Obesity Summit, 4th in the series of obesity-related annual events organized by EuroSciCon, was held from 12 to 14 April 2016 at Cineworld, The O2 in London. This conference set the stage for three days of stimulating high-quality presentations on the advancements in obesity in an informal academic setting. Approximately 156 delegates including students, researchers, healthcare professionals and scientists from 36 countries around the world attended the event. This meeting report summarizes some of the most outstanding presentations.

## Discussion

The Obesity Summit 2016, attracting an audience from 36 countries, commenced with knowledge-driven resounding performances. These included obesity etiology and pathogenesis, rediscovering symptoms, lipidomics, metabolomics, psychological and genetic aspects, fetal reprogramming, as well as confronting challenges to prevent, treat and manage obesity and to restore health consciousness among the general population. The inevitability of creating awareness through aggressive media campaigns, underlining enormity and suggesting dietary; surgical; and physical activity; and sociable pathways was acknowledged. Every presentation reflected deep commitment to annihilate a potentially dangerous illness that would save the world from superfluous health costs. Healthcare professionals presented clinically and academically tested ideas, advancement in surgeries, innovative treatment options, weight stigmas and eating patterns, and strengthened the association between poverty and obesity.

The first day, themed ‘Etiology and Pathogenesis’, opened with an introduction by the chair, Dr Jude Oben (UCL Institute of Liver and Digestive Health, Royal Free Hospital, London, UK). Dr Adrian Vella (Mayo Clinic, NY, USA) discussed the mechanisms of glucose lowering after bariatric surgery. Calorie restriction improves glucose metabolism and plays a significant role in controlling blood glucose and remission in diabetics after bariatric surgery. Several years after Roux-en-Y gastric bypass in nondiabetic subjects, the elevated postprandial GLP-1 concentrations affect insulin secretion and gastrointestinal motility [1]. Professor Michael Symonds (University Hospital, Nottingham, UK) discussed the significance of brown adipose tissue (BAT) as an anti-obesity target and “The Fat that makes you thin”. Factors promoting BAT include birth, stress, diet and exposure to cold. There is a negative relationship between BMI percentile and both baseline supraclavicular temperature (T_SCR_), collocating with the primary region of BAT, and the alteration in T_SCR_ in response to the cool stimulus. He recommended future work to determine what receptors to target for BAT; a class of β3-adrenergic receptor agonists called mirabegron that has been shown to stimulate BAT thermogenesis in humans [2]. The question remains whether we can find a dietary ingredient that could increase the activation of BAT. Professor Timothy Frayling (University of Exeter Medical School, UK) gave a stimulating talk on the paradox concerning genes and obesity. He pointed toward the role of genetics and molecular mechanisms involved in predisposing a person to a higher BMI with a lower risk of obesity-related diseases.

The second day's theme was ‘Psychological Studies’, chaired by Professor Fary Cachelin (University of North Carolina, USA). She presented an overview of the cultural and ethnic perspectives on binge eating, body image and obesity. She concluded that Latinas have higher rates of obesity as compared with white women, with the majority not seeking any professional help. Cultural adaptation along with self-help and cognitive behavior therapy is known to be beneficial. Increasing awareness, promoting treatment and developing effective tailored interventions based on cultural considerations can help curtail eating disorders. Dr Lesley Gray (University of Otago, Wellington, New Zealand) acknowledged the already known, that obesity rates in New Zealand are high, healthcare appears poorly equipped to meet current and expected future needs for very large patients, and these high obesity rates are both physically and socially challenging for health professionals. She outlined a trilogy of research currently being undertaken by Gray and Hales. The first concerns the effect of bariatric simulation suites on perceptions and beliefs of health professionals toward obese people in training environments. The second considers whether there are issues for manual (safe) handling procedures in health care when working with bulky patients and very large equipment. The third asks how can we support health professionals to feel more comfortable discussing issues involving obesity in healthcare settings?

Dr Geraldine McLeod (University of Otago, New Zealand) focused on examining the link between obesity and poverty using latent variable structural equation modeling. She observed that those who were socioeconomically disadvantaged and already obese or overweight by the age of 30 years old had a higher risk of gaining more weight by the time they were 35 years old. She concluded by suggesting that focus should primarily be on trying to prevent weight gain instead of just trying to lose weight. Socioeconomic disadvantage, being a stronger predictor of overweight and obesity than life stress, also needs to be reduced. Hence, improving socioeconomic conditions would minimize obesity, combined with efforts directed toward maintaining the current weight.

Dr Van-Hout (Catharina Hospital Obesity Centre, The Netherlands) talked about interdisciplinary care and psychological aspects of bariatric surgery. He stressed that bariatric surgery is better than untreated obesity. He mentioned obesity as a partial behavior problem, with obesity surgery being a forced behavior modification. Hence, psychological screening, preoperative and postoperative psychological care of patients is essential in improving compliance and adjustments to improve results of bariatric surgery.

‘The Sweet Debate to Lose Weight’ was an interactive question-answer format presentation by Dr Adeel Ahmad (King Faisal Specialist Hospital & Research Centre, Jeddah, Saudi Arabia). He presented the audience with his forceful argument of wakefulness to limit intake of free and added sugar, read food labels with caution and comprehend the perils of eating certain fruits rich in natural sugars. Dr Adeel dwelled on the risks of using different types of sweeteners, their use, impact, implications in weight loss [3], and absence of a clear link to cancers in humans [4]. He emphasized the necessity of productive debate while considering insinuations about the use of brown sugar, honey and other artificial sweeteners. He concluded by highlighting the efforts by Public Health England in reducing sugar intake [5] and concluded his talk by stating the fact that “sugar by any other name, is still sugar”.

Dr Alper Celik (Turkish Metabolic Surgery Foundation, Istanbul, Turkey) chaired the morning session of the final day themed ‘Prevention, Treatment and Management’. He talked about digestive adaptation and metabolic surgery and described the difference between obesity surgery and metabolic surgery. He based his presentation on the concept of “malabsorption free surgery for Type 2 diabetes”. He introduced the idea of intestinal and gastric satiety, functional restriction and a new balance between proximal and distal gut [6]. He mentioned surgical options including ileal proximalization without significant malabsorption, Ileal transportation transposition (with diverted sleeve) and transit bipartition surgeries being effective in achieving functional restrictions. Dr Edward Laskowski (Mayo Clinic, USA) highlighted the significance of integrating high-intensity interval training (HIIT) into an exercise plan for obesity and also described the beneficial effects of HIIT training. Improving PA in a population brings many benefits, including decreasing hospital admissions, lowering healthcare costs and reducing sick leave. Increased sitting time/sedentary lifestyle has been shown to increase metabolic risk factors. Dr Laskowski discussed multiple exercise studies, which show the small, independent effect of exercise on body weight, usually less than 3%. He also stressed that HIIT is an effective and efficient training modality in diverse populations, including those with diabetes, cardiovascular disease or obesity. To reduce weight regain, sustain weight loss and curtail the harmful effects of obesity, most studies suggest that 300 minutes of moderately intense exercise per week is necessary. Dr Laskowski suggested strength training increases lean muscle mass with metabolic changes that cause a reduction in blood pressure, harmful lipids and insulin resistance. Finally, Dr Laskowski recommended a paradigm shift in public health comprising a renewed focus on improving physical activity in the population.

Dr Ellen Govers (Dutch Knowledge Centre for Dietitians on Overweight and Obesity, The Netherlands) produced evidence for diet- and lifestyle-based obesity treatment that would improve comorbidities and quality of life. Food high in protein but low in carbohydrates can reduce eating between meals, facilitate self-restraint and prolong satiety for hours. In 2014, well over 1.9 billion adults were reported overweight worldwide, out of which 600 million were obese. Type 2 diabetes, gout and hypertension are the consequences of deteriorating metabolism owing to insulin resistance [7]. Notably, what we select to eat has a significant bearing on the growth of insulin resistance. For example, saturated fats, excessive carbohydrates and deep-fried foods lead to insulin resistance [8]. She recommended a low-carbohydrate and high-protein diet, more exercise and reduction of stress, supported by specialized healthcare coaches or dieticians.

The three day summit concluded with the announcement of the poster prize winners: Mr Adam Olichwier (Lipid metabolism in hypothyroid heart – the role of stearoyl-CoA desaturase 1) and Professor Akira Sasaki (Effect of laparoscopic sleeve gastrectomy on nonalcoholic steatohepatitis in Japanese patients with severe obesity).

Conclusively, obesity is an unchecked epidemic associated with poverty, inactivity, fatty food consumption and emotional eating. Excessive carbohydrates and saturated fats have a substantial bearing on the promotion of insulin resistance. Since psychological barriers, genetic and environmental factors play a significant role in promoting obesity, awareness campaigns to educate the general population about the perils of overeating and consuming excessive free sugars, and the need to implement physical activity into their daily schedule are of paramount importance. Surgical options to treat obesity – considered as the only effective long-term weight-loss solution – include ileal-proximalization, transit-bipartition, resleeve gastrectomy and bariatric surgery. Trimming of bulky frames is a challenge for all. Health professionals can motivate individuals to overcome cultural sensitivities, compulsive eating, psychological obstructions and social impediments to lose weight, curtail harmful effects of obesity and lead an active life. Since craving for more is a neurotic veracity, trimming of fatty frames and sustaining it in the long-term is a topic for further research and experimentation.
